# EP Fellows Summit 2024: Case Competition Finalists

**DOI:** 10.19102/icrm.2025.16035

**Published:** 2025-03-15

**Keywords:** Cardiac pacing, His-bundle pacing, left bundle branch area pacing

## Abstract

The 2024 EP Fellows Summit & Arrhythmia Scholars Program was held November 1–3, 2024, in Arlington, Virginia.

## Abstract 1

### Atrial Fibrillation Trigger Masked by General Anesthesia

Sang Lee, MD,^1,2^ Joonhyuk Kim, MD,^1,2^ and Jim Cheung, MD^1,2^

^1^New York Presbyterian Queens Hospital, Flushing, NY, USA

^2^New York Presbyterian Weill Cornell Medical Center, New York, NY, USA

The authors report no conflicts of interest for the published content. No funding information was provided.

Corresponding author: Sang Lee, MD; sangslee24@gmail.com

***Background:*** In the United States, more than 454,000 individuals are hospitalized each year with atrial fibrillation (AF) as a primary or secondary diagnosis. AF is responsible for approximately 158,000 deaths annually, and its mortality rate has been on the rise for more than two decades. Rhythm-control therapies, including cardioversion and catheter ablation, have gained prominence in the last decades. Recent studies and large registries indicate that early catheter AF ablation can improve both mortality rates and quality of life, particularly for patients with heart failure. Previous studies suggest that up to 90% of AF triggers originate from the pulmonary veins (PVs), while non-PV triggers can be found in various sites, including the left atrial posterior wall, left atrial appendage, ligament of Marshall, coronary sinus, superior vena cava (SVC), and crista terminalis.

***Case:*** A 62-year-old man with a history of paroxysmal AF (PAF) was referred to the electrophysiology clinic for episodes of non-prodromal syncope and palpitations. An event monitor revealed AF with conversion pauses. He subsequently underwent implantation of a permanent pacemaker and was started on metoprolol. He had no recurrent syncope but continued to report palpitations. In-office device interrogation (IODI) showed a 29% AF burden. He was hesitant on pulmonary vein isolation (PVI) and was started on amiodarone. Due to persistent palpitations, he underwent PVI, after which he initially felt improved. However, he experienced recurrent palpitations, and IODI showed frequent PAF. A second PVI was performed, revealing reconnection of both left PVs. No AF triggers were found, and the left PVs were re-isolated. Despite these interventions, subsequent IODI showed continued PAF. A trial of dofetilide and sotalol was ineffective at separate times. Due to more frequent symptoms, a third PVI was done; all PVs remained to be isolated. Non-PV AF triggers could not be found despite the use of isoproterenol. AF was induced by rapid atrial pacing, and the left atrium was mapped for spatial and temporal dispersion; these areas were ablated with no improvement.

Finally, a fourth PVI was done with moderate sedation as opposed to general anesthesia, which was used in all previous PVIs. Immediately after isoproterenol administration, AF triggers were repeatedly detected from the SVC **(Figure 1A)**. A deep myocardial sleeve was noted in the SVC, and it was isolated. This resulted in a return to sinus rhythm in the atria, whereas the SVC remained in AF **(Figure 1B)**. The patient has since been asymptomatic, with no recurrence of AF for 16 months.

**Figure 1:**
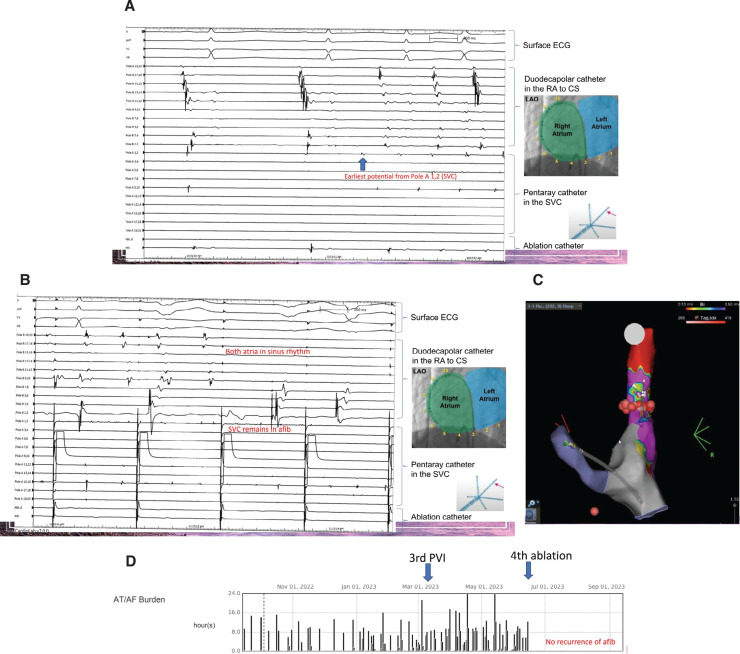
**A:** Electrogram showing the earliest potential from the superior vena cava (SVC). **B:** Electrogram showing the termination of atrial fibrillation during ablation of the SVC. **C:** Mapping of the SVC showing the deep myocardial sleeve. **D:** Remote permanent pacemaker interrogation showing the termination of atrial fibrillation after the fourth ablation (SVC ablation). *Abbreviations:* AT/AF, atrial tachycardia/atrial fibrillation; CS, coronary sinus; ECG, electrocardiogram; LAO, left anterior oblique; RA, right atrium; SVC, superior vena cava.

***Discussion:*** The success rate of radiofrequency AF PVI is up to 85%. Non-PV triggers could be found at subsequent ablations after initial PVI. A routine empiric SVC ablation in all AF cases remains a topic of debate, given the potential complications, such as SVC stenosis, sinus node injury, and right phrenic nerve injury. Additionally, the use of general anesthesia can obscure the identification of non-PV triggers despite the use of isoproterenol and pacing maneuvers.

***Conclusion:*** This case emphasizes the importance of identifying the AF triggers when empiric PVI proves ineffective. It also highlights how the type of anesthesia can impact the detection of non-PV triggers, suggesting that using moderate sedation may improve the success rates of ablation procedures following initial PVI.

## Abstract 2

### An Unusual Cause of Syncope: Right Coronary Vasospasm Leading to Atrioventricular Block

Maxwell D. Coll, MD,^1^ Andres Felipe Miranda-Arboleda, MD,^1^ Victor Nauffal, MD,^1^ Ajar Kochar, MD,^1^ Usha Tedrow, MD, MS^1^

^1^Brigham and Women’s Hospital, Boston, MA, USA

The authors report no conflicts of interest for the published content. No funding information was provided.

Corresponding author: Maxwell D. Coll, MD; mcoll@bwh.harvard.edu

***Background:*** Bradycardia and conduction disorders are well-known sequelae of significant coronary artery disease (CAD). Branches of the right coronary artery (RCA) provide the major blood supply to the sinoatrial node and atrioventricular (AV) node. Transient AV block occurs commonly in the context of acute myocardial infarction. Syncope due to symptomatic AV block resulting from RCA vasospasm is rare.

***Objective:*** This study sought to highlight RCA vasospasm-induced AV block as a potential cause for recurrent syncope and the management considerations for such patients.

***Case:*** A 74-year-old woman with a prior medical history of CAD with recurrent syncope over 3 weeks underwent placement of a drug-eluting stent (DES) to the mid-left anterior descending artery (LAD) without resolution of symptoms. One week following DES placement, she suffered another syncopal event with telemetry demonstrating acute ST-segment elevation in lead II with a 2:1 AV block.

She underwent a coronary angiogram, which demonstrated patent DES in the mid- and distal LAD. A temporary pacing wire was then placed, and she underwent vasospasm testing with acetylcholine delivered to the RCA. Prominent RCA vasospasm was noted **(Figure 1)**, leading to inferior ST-segment changes and progression to AV block requiring temporary pacing until the resolution of vasospasm and return to normal sinus rhythm (NSR). Of note, while pacing improved hemodynamics during vasospasm, she was still hypotensive to 90/50 s until the return to NSR. She was started on isosorbide mononitrate with her home dose of amlodipine but was unable to tolerate higher doses of nitrates due to headaches. Prior to discharge, she underwent placement of a dual-chamber permanent pacemaker to support her heart rate.

**Figure 1:**
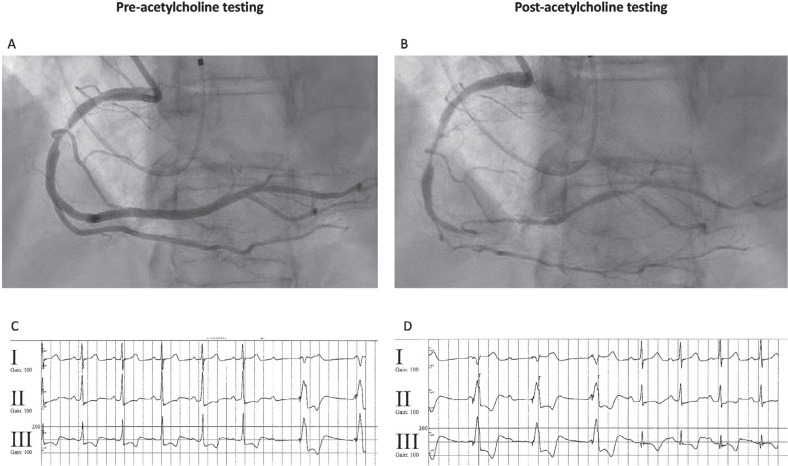
**A:** Right coronary artery pre-acetylcholine testing. **B:** Right coronary artery post-acetylcholine testing. **C:** Onset of vasospasm leading to atrioventricular block requiring initiation of temporary pacing at a backup rate of 60 bpm. **D:** Resolution of vasospasm with return to normal sinus rhythm.

***Conclusion:*** RCA vasospasm can induce transient symptomatic AV block. Syncope may not be solely related to AV block but can also result from vasospasm-induced right ventricular dysfunction. Care should be taken to assess the etiology of hemodynamic compromise to determine if permanent pacing would provide benefits.

## Abstract 3

### Ventricular Tachycardia Ablation with Stacked Pulsed-field and Radiofrequency Energy via a Dual-energy Lattice-tip Catheter: A Novel Two-pronged Approach

James Mannion, MBBCh, BAO, MRCPI^1,2^ and Jonathan Lyne, FESC, FHRS^1,3^

^1^Electrophysiology Department, Beacon Hospital, Dublin, Ireland

^2^Cardiology Department, Cork University Hospital, Cork, Ireland

^3^School of Medicine, University College Dublin, Dublin, Ireland

Prof. Lyne has received honoraria and speaking fees from Medtronic. The other author reports no conflicts of interest for the published content. No funding information was provided.

Corresponding author: James Mannion, MBBCh, BAO, MRCPI; jamesm2016@gmail.com

***Introduction:*** The role of pulsed-field (PF) energy in the ablation of ventricular tachycardia (VT) is not yet well established, with mixed short-term outcomes in initial clinical data as a single energy source.^1,2^ Despite this, PF ablation does show potential promise in terms of ventricular lesion size compared to radiofrequency (RF) ablation.^3–6^ PF lesion dimensions also increase with recurrent applications.^7^ PF energy can also be applied over pre-existing RF lesions with deeper penetration when compared to secondary superimposed RF energy.^8^ In this case, we ablated an ischemic VT using a dual-energy lattice-tip mapping and ablation catheter by superimposing energy sources to areas of interest.

***Case:*** A 60-year-old man with severe left ventricular (LV) dysfunction from ischemic cardiomyopathy with a cardiac resynchronization device and defibrillator presented with recurrent presyncope and collapse. When his device was checked, we identified 51 runs of non-sustained VT, either self-terminating or requiring anti-tachycardia pacing therapy. He did not report any recent chest pain, and high-sensitivity troponin was not elevated. There was no clinical or echocardiographic evidence of volume overload.

Medication included 75 mg of aspirin once daily (OD), 80 mg of atorvastatin OD, 24/26 mg of sacubitril/valsartan twice daily, 5 mg of bisoprolol OD, 10 mg of dapagliflozin OD, 12.5 mg of spironolactone OD, 60 mg of isosorbide mononitrate OD, 40 mg of pantoprazole OD, 150 mg of venlafaxine OD, and 500 μg of dutasteride OD.

He received intravenous amiodarone loading therapy but experienced further uncontrolled symptomatic monomorphic VT, now at a rate of 115 bpm, below the device VT monitoring zone **(Figure 1)**, with the likely VT exit site being the inferior wall of the left ventricle. No identifiable reversible causes were found. We discussed treatment options, and it was agreed to perform a VT ablation. Informed consent was obtained from the patient for the use of this catheter for the procedure.

**Figure 1:**
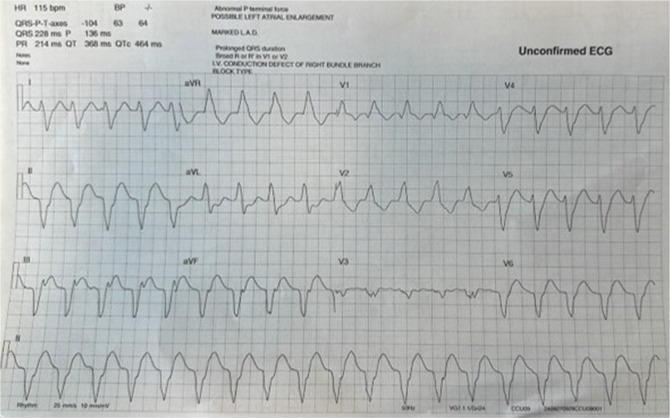
Clinical monomorphic ventricular tachycardia, with the exit site on the left ventricular inferior wall, in keeping with previous ischemia.

The procedure was performed off anti-arrhythmic therapy via a retrograde approach under general anesthetic, with heparin administration via activated clotting time guidance.

The left ventricle was mapped with a dual-energy lattice-tip mapping and ablation catheter (Sphere-9 Catheter, Affera Mapping and Ablation System; Medtronic Inc., Minneapolis, MN, USA). Bipolar voltage mapping was performed with <0.5 mV as a low-voltage surrogate for LV scar. Large areas of scar were noted on the inferior/inferolateral walls **(Figure 2)**. While being biventricularly paced, areas of late activation and fractionation were identified related to the scar **(Figure 3)**.

**Figure 2:**
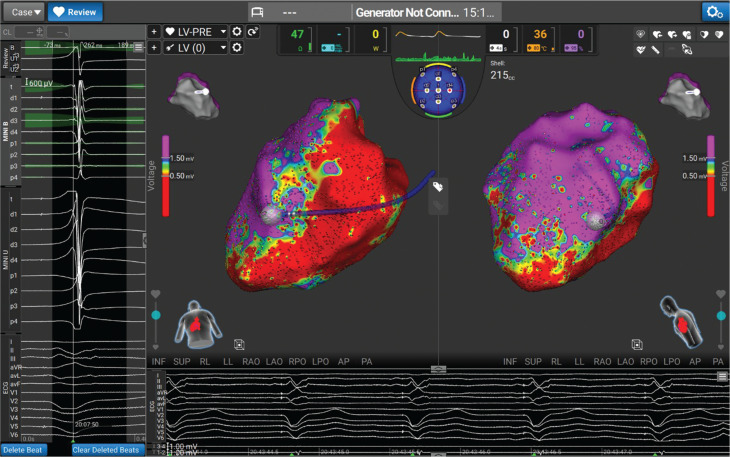
Bipolar voltage map of the ventricle, with large territory of the scar (<0.5 mV) on the inferior wall.

**Figure 3:**
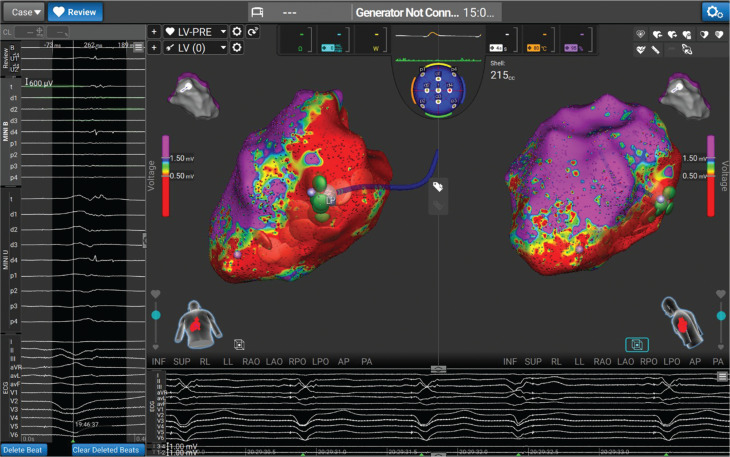
Areas of late potential and fractionation during biventricular pacing, and the target for our dual-energy ablation.

The VT was inducible but non-sustaining, so comprehensive tachycardia activation mapping or entrainment could not be performed despite several attempts. Instead, areas of fractionation and late activation during biventricular pacing were targeted, as shown in **Figure 3**. RF was used in the first instance, and, when lesions were complete, the energy was toggled to PF, with further repetitive superimposed applications as demonstrated in **Figure 4**.

**Figure 4:**
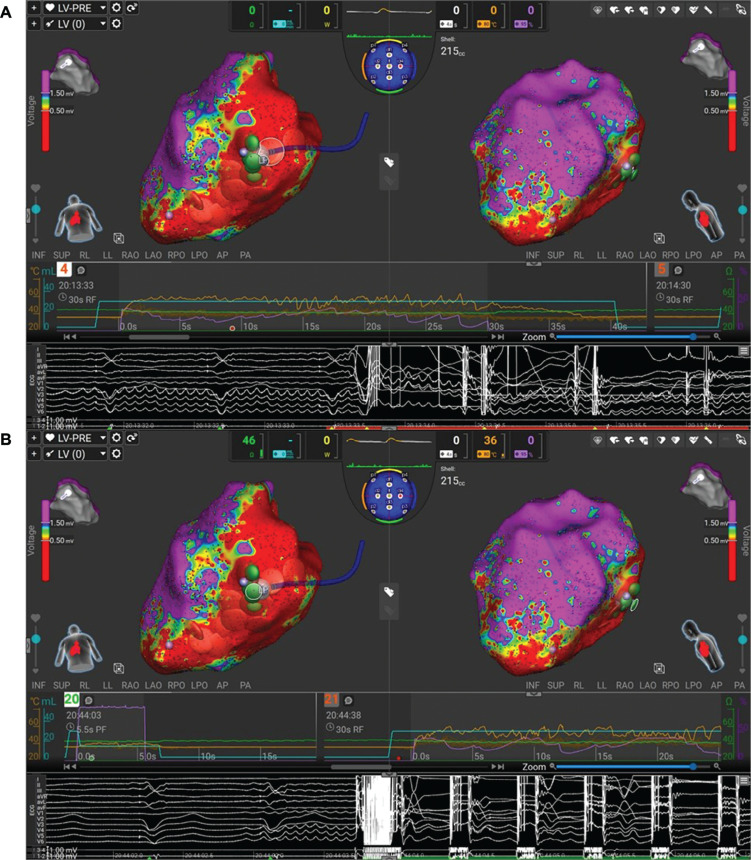
Radiofrequency **(A)** and subsequent pulsed-field energy **(B)** being applied to the areas of interest (marked late potential), with sequential applications compounding upon each other.

After ablation, the clinical VT could not be induced. There were no areas of late activation remaining in the scar region after the procedure. There were no acute complications, and post-procedural echo did not demonstrate any new effusion or LV dysfunction. The patient was admitted to the intensive care unit for monitoring after the procedure.

***Discussion:*** Currently, RF is the most well-studied energy source in ablation involving the left ventricle; however, PF has several proven preclinical advantages, with further theorized benefits. Initial clinical data from small cohorts where VT ablation is performed with PF used a single energy source have yielded mixed results with alternative catheters.^1,2^ PF energy, however, does show promise in generating lesions that are deeper and larger than those generated via RF in computational^4^ or animal models.^3,5,6^ Repetitive PF applications also result in increased lesion dimensions.^7^

This novel catheter (Sphere-9 catheter) currently has a CE certificate for atrial arrhythmias only. There has been an example of its use, however, in a patient like ours with ischemic cardiomyopathy undergoing ventricular ablation.^9^ This patient had RF and then PF energy applications, and there was no recurrence of arrhythmia by 5 months.

PF applications may be superimposed over chronic RF lesions, and this can modify further additional substrate, penetrating deeper through areas of heterogeneous fibrosis than RF.^5^ Using dual superimposed energy sources in this way may compound on the results of RF and further improve lesion homogeneity, depth, and size compared to RF alone.

***Conclusion:*** Although the role of PF energy in ventricular ablation is not fully established, using acute RF with superimposed PF applications is feasible. This may result in increased lesion depth and size, especially with compounding applications. Our patient demonstrated acute markers of procedural success, as we were unable to induce the clinical VT, and there were no acute complications.
